# Crohn’s disease, irritable bowel syndrome, and chronic fatigue: the importance of communication and symptom management—a case report

**DOI:** 10.1186/s13256-024-05010-3

**Published:** 2025-01-09

**Authors:** Johannes Haedrich, Roman Huber

**Affiliations:** Center for Complementary Medicine, Department of Internal Medicine II, Faculty of Medicine, Medical Center – University of Freiburg, University of Freiburg, 79106 Freiburg, Germany

**Keywords:** Autonomic nervous system, Gut-brain axis, Complementary medicine, Opium, Somatoform pain

## Abstract

**Background:**

Crohn’s disease and irritable bowel syndrome may both cause abdominal pain and diarrhea. Irritable bowel syndrome not only is an important differential diagnosis for Crohn’s disease but also occurs in one out of three patients with Crohn’s disease in remission in parallel. If not adequately diagnosed and treated, additional functional symptoms such as fatigue and/or muscle pain may develop, indicating a more severe course.

**Case presentation:**

A 64-year-old Caucasian male with long-standing, widely inactive Crohn’s disease presented with persistent diarrhea, bloating, abdominal pain, general fatigue, unexplained hip pain, and frequent shivering with cold extremities, which had worsened following a gastrointestinal infection and psychological stress. A plausible explanation of his symptoms, based on an understanding of mind–body interactions, the autonomic nervous system, and temperature regulation, combined with symptom relief, was associated with rapid and sustainable improvement. After 2.5 years of follow-up, the patient is almost symptom-free.

**Conclusions:**

This case report exemplifies the interrelation between organic (Crohn’s disease) and functional diseases (irritable bowel syndrome, chronic fatigue syndrome, and somatoform pain). It further demonstrates that these connections may be overlooked in daily practice and that providing a plausible explanation in combination with symptom relief may be important for patients with functional syndromes.

## Background

Inflammatory bowel diseases (IBDs) include the subtypes Crohn’s disease (CD) and ulcerative colitis (UC) and are characterized by an overactive immune system destructive to the gastrointestinal tract [1–3]. Patients with CD present with various, often unspecific abdominal symptoms, in particular diarrhea, bloating, cramps, abdominal pain, and fatigue. In more severe cases, bloody stools, weight loss, fever, anemia, stenosis, or fistulae may be present [4, 5]. Owing to overlapping symptoms, irritable bowel syndrome (IBS) is an important differential diagnosis for CD. IBS is considered a common functional disorder of gut–brain interaction, with symptoms mainly triggered by diet, stress, medications, and gastrointestinal infections [6–11]. It is now established that not only IBS but also IBD is associated with dysregulated gut–brain and microbiota–gut–brain axes, providing the physiological link between the central nervous system (CNS) and the gastrointestinal tract [12–16]. Accurate diagnosis, primarily through ileocolonoscopy and blood tests, is essential to distinguish between these two conditions and other possible disorders, ensuring appropriate management. The treatment of CD aims to control the body’s inflammatory response with the help of immunomodulators and corticosteroids. Surgical intervention may be required in case of stenosis or fistulae.

Over the past two decades, a growing body of evidence has shown that patients with CD in remission may experience symptoms of IBS [17–25], sometimes making differentiation challenging, as concomitant IBS symptoms can mimic active IBD. Accurate diagnosis through patient-centered dialog is crucial for appropriate medical and behavioral management [18, 26–30]. Studies have shown that the frequency of IBS in CD patients in long-term remission can be as high as 35%, which is three times higher than the global prevalence of IBS symptoms in the general population (11%) [8, 17–25], with a considerable impact on their quality of life [19, 20]. With this case report, we aim to highlight this concurrence of CD with IBD and provide an example of initial neglect and ultimately successful treatment.

## Case presentation

### Current medical history

A 64-year-old Caucasian male presented in February 2022 with persistent diarrhea, bloating accompanied by abdominal pain, frequent headaches, shivering, and cold extremities lasting for 2–3 weeks each month, insomnia, and general fatigue. He had a history of Crohn’s disease (CD) with an ileocecal resection involving 42 cm of the terminal ileum and 10 cm of the cecum immediately following his initial diagnosis in early 1981, Montreal Classification A2, L3, B3 [31]. Biopsies from the terminal ileum and ascending colon revealed transmural inflammation and epithelioid granulomas. Since surgery, CD had widely remained in remission, with occasional episodes of low-grade nonspecific ileitis (1984, 1991, 2006) and low-grade chronic colitis (1986) seen on ileocolonoscopy (Table 1). Inappropriate to CD in remission, the patient suffered from chronic diarrhea, up to four times a day and often imperative, which numerous medical specialists that he consulted considered to be unusual. Annual ESR, CRP, and blood count results were normal. The patient was taking cholestyramine 6 g per day, loperamide hydrochloride 2 mg per day, probiotic bacterial cultures (1.1 × 10^10^ CFU per capsule) two capsules once daily (self-medication), cyanocobalamin 1000 µg IM every 6 months, and ramipril 5 mg once daily. He had no history of smoking or alcohol consumption and reported a paternal family history of asthma and neurodermatitis. There was no family history of inflammatory bowel disease (IBD) or irritable bowel syndrome (IBS).

**Table 1:** Timeline: patient’s symptoms, diagnoses, treatment, and significant events, based on available medical reports and patient’s self-disclosure

Date	Age (years)	Symptoms	Diagnoses	Treatment	Significant events, remarks
10/1957–04/1958	0–1/2	Diarrhea at age 4–12 weeks	Unknown	11/1957: Breast feeding ends at age 6 weeks (diarrhea)	After birth, patient is transferred to a baby nursing home for 6 months; reportedly crying most of the time, refusing food
1958–1966	1–9	frequent colds, otitis media, coughing; eczema from red currants and rhubarb	Asthma, dermatitis	Antibiotics 02/1966: Tonsillectomy; 04/1966: 6 weeks at a North Sea sanatorium	04/1966: Patient admitted to children’s sanatorium on the North Sea for 6 weeks (“deported child”) where he like many other children suffers psychological and physical violence
10–12/1980	23	Diarrhea 4–6/day; intermittent, then abdominal pain Weight loss 16 kg within 4 months (now 62 kg, 176 cm)	Duodenal ulcer (suspected)	19 Dec 1980: Cimetidine 200 mg PO qid	Pre-graduate exams (chemistry); persistent diarrhea, abdominal pain after food intake; symptoms alleviate during physical exercise. OGD, U/S; ESR, complete blood count and all other laboratory results normal
02/1981	23	Diarrhea 6–8/day; increasing pain in right lower abdomen; body temp. > 39 °C (102 °F)	Crohn’s disease, ileocolitis, iliopsoas abscess	Exploratory open surgery, iliopsoas abscess drained; Open abdominal surgery, ileocecal resection Sulfasalazine 2 g PO qd	26 Feb 1981: Open abdominal surgery, conglomerate of caked intestinal loops removed: 42 cm terminal ileum, 10 cm cecum; histology: transmural inflammation, epithelioid granulomas
03/1981–02/1982	23–24	Diarrhea 3–4/day	Crohn’s disease, in remission; chologenic diarrhea	Sulfasalazine 2 g PO qd	03/1981: Parenteral diet; weight 56 kg 04/1981: Patient resumes studies while still hospitalized 05/1981: Wt 60 kg Diet: Oatmeal, toast, rusks, banana, white rice, steamed potatoes, carrots 10/1981: Outpatient examination at Freiburg University Medical Centre. U/S, X-ray Sellink, ESR, complete blood count and all other laboratory results normal; weight 65 kg Semi-annual ESR and complete blood count results are normal
03/1982	24	Diarrhea 0–1/day	Crohn’s disease, in full remission; gallstones, chologenic diarrhea	Cholestyramine 2 g PO tid	U/S, X-ray Sellink, ESR, complete blood count and all other laboratory results normal; weight 67 kg
09/1984	26	Diarrhea 3–4/day	Crohn’s disease, mild activity; gallstones, chologenic diarrhea	Cholestyramine 2 g PO tid; sulfasalazin 2 g PO qd; steroids temporarily	Colonoscopy, histology: slight nonspecific inflammation in ileal mucosa U/S, X-ray Sellink, ESR, complete blood count, and all other laboratory results normal. BM 4–5/day; weight 67 kg
07/1985–10/1985	27	None	Crohn’s disease, in full remission; chologenic diarrhea	Cholestyramine 2 g PO tid; loperamide HCl 2 mg PO qd (self-medication); cyanocobalamin 1000 µg IM q3mos	Low vitamin B12; weight 67 kg Trial and error-based vegetarian diet, for details see “Medical and lifestyle interventions” section
06/1986	28	Diarrhea 3–4/day; upper abdominal pain	Crohn’s disease with low-grade chronic colitis; chologenic diarrhea; gastroduodenitis	Cholestyramine 2 g PO tid; loperamide HCl 2 mg PO qd; cyanocobalamin 1000 µg IM q3mos; steroids temporarily	Colonoscopy, histology: low-grade chronic colitis, low-grade chronic proctitis. OGD: moderate acute and chronic gastro-duodenitis U/S, X-ray Sellink, ESR, complete blood count and all other laboratory results normal; weight 64 kg
07/1986–08/1990	28–32	Diarrhea 1–2/day; upper abdominal pain	Crohn’s disease, with low-grade chronic colitis; chologenic diarrhea	Cholestyramine 2 g PO tid; loperamide HCl 2 mg PO qd; cyanocobalamin 1000 µg IM q3mos	Semi-annual ESR and complete blood count results are normal, weight 64–67 kg Patient consults various gastroenterologists as to why diarrhea persists even though Crohn’s disease is in remission. General response: Reasons are unknown but your condition should actually be better 04/1989: Patient accepts position at a Federal Food Investigation Agency
01/1991	33	Diarrhea 4–6/day; upper abdominal pain	Crohn’s disease with low-grade ileitis; cholecystolithiasis; chologenic diarrhea;	Continued	Colonoscopy, histology: low-grade nonspecific ileitis U/S, ESR, complete blood count and all lab results normal except elevated ALT, AST, and gammaGT, BM 4–6/day; weight 67 kg The more frequent diarrhea was explained by bile acid loss syndrome and a stress-related functional component
02/1992–07/1995	34–37	Diarrhea 1–3/day; upper abdominal pain	Crohn’s disease in remission; cholecystolithiasis; chologenic diarrhea	Continued	04/1992: Laparoscopic cholecystectomy Semi-annual ESR and complete blood count results are normal. BM 3–4/day; weight 70 kg
10/1995–06/2006	38–49	Diarrhea 1–2/day	Crohn’s disease, in remission; chologenic diarrhea	Continued	Semi-annual ESR and complete blood count results are normal. BM 3–4/day; weight 72 kg Patient consults various gastroenterologists as to why diarrhea persists even though Crohn’s disease is in remission. General response: Reasons are unknown but your condition should actually be better
07/2006–10/2007	49–50	Diarrhea 2–4/day; more often cold symptoms	Crohn’s disease with low grade ileitis; chologenic diarrhea; lactose intolerance	Continued 09/2006: Prednisolone 20 mg PO qd × 4 weeks, then reduced weekly by 2.5 mg PO qd	07/2006: Patient assumes a demanding position at a European Union research institution Semi-annual ESR and complete blood count results are normal. BM 3–4/day; weight 72 kg 09/2006: Colonoscopy, histology: low-grade nonspecific ileitis 05/2007: Abdominal MRI: normal findings. Lactose intolerance test: positive result
2008–2011	49–55	Diarrhea 1–2/day	Crohn’s disease in remission; chologenic diarrhea; lactose intolerance	Cholestyramine 2 g PO tid; loperamide HCl 2 mg PO qd; cyanocobalamin 1000 µg IM q6mos	Patient’s work is highly demanding. Patient is bullied by a superior Annual ESR and complete blood count results are normal. BM 3–4/day; weight 74, increasing to 76 kg
2012–2016	54–59	Diarrhea 1–2/day	As before, new diagnosis: hypertension	Continued, in addition ramipril 5 mg PO qd;	05/2012: Colonoscopy: no signs of inflammation in neoterminal ileum and colon Patient is increasingly bullied by a superior Annual ESR, CRP, and complete blood count results are normal. BM 3–4/day; weight 76, increasing to 80 kg
2017–2018	60–61	Diarrhea 2–3/day, belly pain; 6 × annually flu-like symptoms for 2–3 weeks, general fatigue, urinary urgency	As before	Continued	Patient feels hardly sociable, often cannot keep appointments; takes light diet. Bullying by superior intensifies in 01–06/2017 07/2017: Medical leave due to deteriorating state of health BM 4–5/day; weight 82 kg
03/2019–01/2022	62–64	Diarrhea 3–4/day, bloating with belly pain; general fatigue, insomnia, flu-like symptoms 1/month for 2–3 weeks, strong unilateral hip joint pain	As before	Continued, in addition self-medication with probiotics	03/2019: Patients suffers an intestinal infection with vomiting diarrhea Frequent flu-like symptoms after exposure to cool air and moderate physical exercise, 1/month for 2–3 weeks: diarrhea and bloating with abdominal pain, general fatigue, insomnia; headache, brain fog, dizziness, poor memory, extreme tiredness (preceding BM); cold extremities, disturbed thermoregulation, urinary urgency, hypertension. Strong, unilateral hip joint pain since 11/2020. Probiotic bacteria trials prove ineffective Patient feels no longer sociable, makes no appointments; takes light diet 08/2019: Colonoscopy: no inflammation in neoterminal ileum and colon 04/2020: Early retirement for health reasons 09/2021 MRT and 11/2021 X-ray: Bilateral age-related low-grade hip osteoarthritis not explaining pain 11/2021: Crohn’s disease activity index (CDAI) [32]: 183 points Patient consults various specialists in gastroenterology, internal medicine, psychosomatics and nutrition as to why his symptoms persist even though Crohn’s disease is in remission. General response: Frequent colds are attributed to a weakened immune system due to stress Annual ESR, CRP, and complete blood count results are normal. BM 4–6/day; weight 85 kg
02/2022	64	Diarrhea 3–4/day, bloating with belly pain, insomnia, general fatigue; flu-like symptoms 1/month for 2–3 weeks; strong bilateral hip joint pain	As before, suspected secondary IBS	Tinctura Opii 6 gtts (3 mg anhydrous morphine) PO bid instead of loperamide. Otherwise continued	Patient consults a specialist in internal medicine, gastroenterology, and complementary medicine Start with multimodal complementary medicine intervention Explanation of symptoms by disturbance of the vegetative nerve system (sympathetic–parasympathetic, gut–brain axis, disturbed thermoregulation) Patient is advised of the following: 1. Tinctura Opii to reduce bowel motility 2. Light dinner should be taken early 3. Moderate physical exercise (HR < 130 bpm, pacing); relaxation techniques Patient experiences a calming positive effect by receiving an explanation for the symptoms experienced for the first time in more than 40 years
03/2022–12/2023	64–66	Diarrhea 1–2/day with bloating on 2–3 days/month; flu-like symptoms 3–4/year for 1–2 weeks; strong bilateral hip joint pain	As before, suspected CFS	As before, in addition lavender oil 80 mg PO bid; 01/2024: 16 mg candesartan cilexetil / 5 mg amlodipine PO qd instead of ramipril; methyl cobalamin 500 µg ODT biw instead of cyanocobalamin	Patient experiences massive improvement in life quality, sociability is regained; flights to North America and Asia for the first time in 5 years Frequency of described symptoms significantly reduced; diarrhea, bloating, abdominal pain still occur after acute stress or physical exertion, and after exposure to cold air. Insomnia, headache, brain fog, dizziness, poor memory, extreme tiredness prior to BM, strong bilateral hip joint pain persists, yet frequency and intensity were noticeably reduced Trial and error-based vegetarian diet, for details see “Medical and lifestyle interventions” section ESR, CRP, complete blood count normal. BM 1–2/day; weight 80 kg
01/2024–06/2024	66	Diarrhea 1–2/day with bloating on 2–3 days/month; flu-like symptoms 3–4/year for 1–2 weeks; mild unilateral hip joint pain	As before	Continued, in addition L-glutamine 5 g PO qd × 1 week, 10 g PO qd × 1 month, then 10 g PO bid	Further stabilization and partial disappearance of symptoms experienced since 03/2019. Diarrhea, bloating may occur after acute stress or physical exertion, now mildly and generally without abdominal pain; insomnia 2–3 days/month; no general fatigue, brain fog dizziness, poor memory or extreme tiredness. Only mild unilateral hip joint pain 06/2024: Crohn’s disease activity index (CDAI) [32]: 87 points ESR, CRP, complete blood count normal. BM 1–2/day; weight 81 kg

### Expanded medical history

From early childhood, he was exposed to stressful life events, including being placed in an infant home from age 0 to 6 months, experiencing physical and emotional abuse in a children’s sanatorium at age 9, and frequent changes of caregivers (various nurses, grandparents, an aunt, parents, and a nanny). Until the age of 3, he frequently refused to eat and later suffered from asthma, food-related skin rashes, and frequent infections, which were treated with antibiotics and resulted in frequent absences from school. At age 9, he underwent tonsillectomy and was sent to a sanatorium 900 km away.

When the patient was 21 (1979), during his first year of studies at university, an elevated ESR was incidentally discovered. Persistent diarrhea began during his pre-graduate examinations in 1980, accompanied by weight loss (from 78 to 62 kg within 4 months) and abdominal pain after food intake (Table 1). In early 1981, the patient canceled his current year of studying abroad owing to persistent diarrhea, increased and persistent pain in the right lower abdomen, continuous weight loss, and general fatigue. When fever occurred, exploratory open abdominal surgery was performed in February 1981 to drain a right-sided iliopsoas abscess. Two weeks later, a conglomerate of caked intestinal loops was removed during an open ileocecal resection (ICR). The patient was discharged on a light vegetarian diet and sulfasalazine 2 g once daily, with a weight of 60 kg. The main symptom remained diarrhea, with up to six episodes out of up to eight bowel movements (BM) per day. Cholestyramine instantly reduced the frequency of diarrhea to 0–2 per day (1–3 BM/day). From 1985 (age 27), the patient’s condition stabilized. He spent 2 months in the Philippines for extensive martial arts training, enjoying very good health with no CD-related symptoms. The patient followed a vegetarian diet but avoided milk, eggs, hard cheese, wholegrain products, raw vegetables, citrus fruits, and fruit juices to prevent bowel problems. In 1995, he underwent laparoscopic cholecystectomy for cholecystolithiasis, a common complication in CD patients (Table 1).

During years of being bullied by a superior (2008–2017) while holding an attractive position at a European Union research institution since 2006, the patient’s blood pressure increased from 130/85 mmHg in 2008 to 160/95 mmHg in 2017, and his weight rose from 74 to 82 kg. Additional symptoms manifested in 2017, primarily painful diarrhea two to three times daily, frequent flu-like symptoms, fatigue, urinary urgency, and cold extremities (Table 1). Flu-like symptoms and shivering mainly occurred after fitness exercise, long-distance flights, or exposure to cold air. They occurred without fever, did not involve the upper respiratory tract, and lasted for 2–3 weeks approximately six times a year. The patient had to take extended medical leave in 2017, ultimately leading to early retirement for health reasons in 2020. He no longer felt sociable and avoided making appointments. He consulted gastroenterologists, internists, and psychosomatic specialists, who explained, that the frequent “colds” were most likely due to a weakened immune system caused by stress. Following a severe gastrointestinal infection of unknown origin in 2019, episodes of diarrhea, bloating with abdominal pain, and flu-like symptoms intensified. These episodes now occurred on a monthly basis especially after exposure to cool air or moderate physical exercise, each lasting 2–3 weeks. They were accompanied by general fatigue, headache, brain fog, dizziness, poor memory, exceptional tiredness, and abdominal pain, which was most pronounced before BMs. Additionally, bloating, sometimes accompanied by diarrhea, tiredness, and drowsiness, would occur within an hour of commencing physical exercise or intense intellectual work, forcing the patient to suspend these activities. Conversely, a short walk or light exercise alleviated headache, fatigue, and tiredness. Insomnia and the inability to wind down resulted in nonrestorative sleep and daytime tiredness.

The patient also reported episodic unilateral hip pain starting in 2020, which became bilateral by 2022, sometimes so severe that he was unable to walk more than 100–200 m. However, MRI and X-ray scans only revealed low-grade osteoarthritis, which did not explain the severe intermittent pain. The patient’s self-trials with probiotic bacteria, aimed at alleviating a suspected leaky gut syndrome, proved unsuccessful. Colonoscopy and histology results in August 2019 showed no inflammation in the neoterminal ileum and colon, suggesting that CD was in remission. This was supported by normal ESR, CRP, and complete blood count results.

### Physical examination and psychological evaluation

The patient presented in good general condition with a normal body mass index (BMI) of 27 (height 1.76 m, weight 85 kg). He had two surgical scars on his abdomen. Bowel sounds were hyperactive, indicating increased peristalsis, and palpation of the abdomen was normal without tenderness. Examination of the chest, joints, spine, muscles, thyroid, and neurological status revealed no relevant pathological findings. His pulse rate was regular at 58 bpm, and his blood pressure was 150/90 mmHg. The patient was clear in his thoughts, emotionally responsive, and exhibited a normal drive with a tendency toward perfectionism.

### Diagnostic interpretation

Our patient presented with a Crohn’s Disease Activity Index (CDAI) score [32] of 183 points (based on weight 85 kg, hematocrit 45%), slightly above the threshold of 150 points, indicating only mild and not necessarily CD-specific activity. The elevated CDAI score was attributable to persistent diarrhea and abdominal pain associated with bloating. Nonspecific, low-grade colitis and ileitis had been detected twice between 1984 and 2006 during regular ileocolonoscopies and histopathological examination of biopsies but did not explain the bowel symptoms that worsened after exposure to cold air, physical exertion, or certain dietary products. Chologenic diarrhea was likely present in the years following ileocecal resection, though not diagnosed specifically, as the patient promptly responded to cholestyramine. Therefore, alternative causes other than CD were considered to explain the patient’s ongoing diarrhea over the past four decades and the additional symptoms experienced since 2017 (Table 2). Food allergies, fructose or lactose intolerance, small intestine bacterial overgrowth, and gluten intolerance were addressed and excluded through detailed history taking or blood tests (negative transglutaminase antibodies). We considered the intermittent pain related to defecation as atypical for CD and more typical for irritable bowel syndrome with diarrhea (IBS-D) [17].

**Table 2:** Course of patient’s symptoms, based on available medical reports and patient’s self-disclosure. CD, Crohn’s disease; IBS-D, irritable bowel syndrome with diarrhea; CFS, chronic fatigue syndrome; ICR, ileocecal resection. Differentiation: CD-like symptoms [2], IBS-D-like symptoms [6, 33], and CFS-like symptoms [49, 85]

Time sections → symptoms (grouped in chronological order of occurrence)↓	Likely attributable to	A 10/1980–12/1980	B 01/1981–02/1981	C 03/1981–02/1982	D 03/1982–06/1985	E 07/1985–06/2006	F 07/2006–12/2016	G 01/2017–02/2019	H 03/2019–02/2022	I 03/2022–12/2023	J 01/2024–06/2024
Abdominal pain after meals	CD	×	×								
Bowel movements	CD, IBS-D	4–6/day	6–8/day	4–5/day	1–3/day^1^	1–4/day^2^	3–4/day^3^	4–5/day	4–6/day	2/day^4^	2/day^4^
Diarrhea	CD, IBS-D	4–6/day	6–8/day	3–4/day	0–2/day^1^	0–3/day^2^	1–2/day^3^	2–3/day	3–4/day	0/day^4^	0/day^4^
Weight loss	CD	×	×								
Abdominal pain, persistent	CD		×								
Fever	CD		×								
General fatigue	CD, IBS-D		×					2–3 weeks/2 months	2–3 weeks/month	1 week/3 months	
Iliopsoas abscess	CD		×								
Ileocolitis (Crohn’s colitis)	CD		ICR	In remission	In remission	In remission	In remission	In remission	In remission	In remission	In remission
Low-grade ileitis	(CD)				09/1984	01/1991	09/2006				
Low-grade colitis	(CD)					06/1986					
Abdominal pain, intermittent	IBS-D							(×)	×	(×)	
Flu-like symptoms	CFS							2–3 weeks/2 months	2–3 weeks/month	1 week/3 months	1 week/6 months
Hypertension	(IBS)^5^							×	×	× ^6^	× ^6^
Urinary urgency	IBS-D, CFS							×	×	×	(×)
Arthralgia (hip joint pain)	CFS								×	×	(×)
Bloating with abdominal pain	IBS-D								2–3 weeks/month	1 week/3 months	(×)
Brain fog, dizziness, poor memory	CFS								×	(×)	
Extreme tiredness	CFS								×	(×)	
Headache	CFS								×	(×)	(×)
Insomnia, unrefreshing sleep	CFS								2–3 weeks/month	1 week/month	2–3 days/month
Post-exertional malaise	CFS								×	(×)	(×)
Temperature dysregulation	CFS								2–3 weeks/month	×	(×)

**A** symptoms onset; **B** severe symptoms; iliopsoas abscess removed, ileocecal resection; **C** first year after surgery; **D** begin cholestyramine intake; **E** begin loperamide intake; **F** assuming a highly demanding position in 07/2006; **G** medical leave in 07/2017; **H** severe gastointestinal infection in 03/2019; **I** complementary medicine intervention at UZN Freiburg in 02/2022; **J** begin L-glutamine intake

^1^Exception in 09/1984: BM 4–5/day, diarrhea 3–4/day

^2^Exceptions in 06/1986: BM 4/day, diarrhea 3–4/day and in 01/1991: BM 4–6/day, diarrhea 4–6/day

^3^Exception in 09/2006: BM 3–4/day, diarrhea 2–4/day

^4^Only on 2–3 days/month: BM 3–4/day, diarrhea 1–2/day

^5^Hypertension has been found to be associated with IBS [86]

^6^Now well under control. (×) symptoms mild, further decreasing and/or less frequent

Hallmarks of diarrhea-predominant irritable bowel syndrome (IBS-D), a type of IBS likely present in our patient, include otherwise unexplained persistent diarrhea, bloating, and abdominal pain occurring at least once a week, according to the Rome IV diagnostic criteria [8, 33]. Factors involved in this disorder of the gut–brain axis include increased intestinal permeability, intestinal sensitivity, increased gut motility, changes in gut microbiota composition, visceral hypersensitivity, stress, and anxiety [6, 10, 15].

The natural course of symptoms and risk factors contributing to IBS can begin as early as infancy [8] (Table 1). Early adverse life events, such as those experienced by our patient, can trigger the development of somatic complaints later in life. Risk factors associated with IBS include loss and separation during childhood [34, 35], parental deprivation [35], or rejection [36], emotional [37, 38] and physical abuse [36–39], punishment [36, 37], conflicted or dependent maternal relationships [34], suppressed feelings of anger and resentment [37], and childhood anxiety [36]. Conversely, studies have shown that parental emotional warmth can be protective against IBS [36]. Frequent infections and antibiotic use in infancy are associated with an increased risk of IBD, while breastfeeding has been shown to be protective against IBD [40].

In adults, stress-induced alterations in neuroendocrine–immune pathways affect the microbiota–gut–brain (MGB) axis, modulated by the vagus nerve, which can lead to a flare-up of IBS symptoms [6, 7, 10, 15, 41]. Stressful life events are known to potentially exacerbate IBD flare-ups by transmitting stress signals from the brain via the enteric nervous system (ENS), triggering inflammatory responses in the gut [42, 43]. Therefore, both IBD and IBS treatment should include the management of stress and stress-related responses [16]. Given our patient’s personal and medical history, and that the symptoms experienced while CD had largely been in remission and without stenosis, we assume that adverse early life events and chronic stress throughout childhood, adolescence, and professional life likely contributed to the development and eventual exacerbation of IBS-D flares.

Increased urinary frequency and urinary urgency, including nocturnal urination, as noted in our patient, have been shown to be associated with IBS and primarily IBS-D, suggesting shared pathophysiology and visceral interactions between the two closely located organs via common sensory pathways [44]. Dysfunction in the autonomic nervous system, that is, an imbalance between the sympathetic and parasympathetic nervous systems, can lead to an overactive bladder [45]. Urinary tract infection, neurological causes, and benign prostate hyperplasia were excluded. Every 2–3 weeks, after physical exertion or exposure to low temperatures, our patient experienced flu-like symptoms and shivering accompanied by diarrhea and cold extremities (Tables 1, 2). Cold extremities can also be related to increased sympathetic nerve activity, as it plays a key role in both circadian temperature regulation and stress response, resulting in peripheral vasoconstriction and consequently reduced heat loss and an increase in the body’s core temperature [46–48].

Following a severe gastrointestinal infection in 2019, the patient experienced additional symptoms including headache, brain fog, dizziness, failing memory, and exceptional fatigue after only light physical activities. These symptoms are reminiscent of a condition termed post-exertional malaise (PEM), a characteristic feature of chronic fatigue syndrome (CFS). Indeed, CFS has been shown to occur more frequently in patients with IBS following intestinal infections [49–53]. Strong mental or physical exertion, or time pressure, exacerbated these neurological and cognitive deficits and caused bloating with abdominal pain within about an hour. Conversely, some relief was observed after prolonged periods of relaxation, often overnight [41, 42]. We assumed that the patient approached these activities with high tension and self-expectations, which could have triggered the mentioned vegetative symptoms. Arthralgia/myalgia manifested in the patient in 2020 and can be interpreted as a further somatoform symptom, belonging to the CFS–fibromyalgia complex. The pain-related functional limitations significantly impaired his ability to participate in daily life. Nonrestorative sleep, tinnitus, urinary urgency, and impaired thermoregulation complete a pattern strongly suggestive of autonomic nervous system dysregulation associated with existing IBS-D (Table 2). Our patient observed symptom flare-ups when swimming in cold water or being exposed to cold air. While experiencing almost continuous flare-ups in winter, symptoms were less intense and occurred less frequently in summer. We interpreted this pattern as consistent with our hypothesis. Cool ambient temperatures would activate the sympathetic tone and trigger bodily stress reactions mediated by the autonomous nervous system in this sensitive individual [54]. A prevalence of thermal hypersensitivity, an altered sensation in response to thermal stimuli, along with elevated cortisol levels, has also been found in IBS-D patients [55, 56]. In another study, IBS patients showed significant symptoms after cold water intake [57]. The severity of IBS symptoms and both visceral and thermal hypersensitivity have been shown to be positively correlated [56].

The severe intestinal infection in 2019 exacerbated the patient’s IBS-D symptoms and triggered new debilitating symptoms (neurological, cognitive), as mentioned above. This deterioration can also be understood within the context of our hypothesis if the intestinal infection is regarded as a threatening, stressful event (Tables 1, 2). Studies have shown that up to 35% of IBS-D patients [58] experience a sudden onset or worsening of IBS symptoms following bacterial or viral gastroenteritis, which is one of the strongest risk factors for IBS [8, 52, 58–60]. We assume that the infectious gastroenteritis affected the gut–brain axis and increased IBS flare-ups in our patient, classifying this as post-infectious irritable bowel syndrome (PI-IBS) [10, 11, 16, 58, 60–65].

In summary, the variety, intensity, and persistence of our patient’s complaints, including numerous nongastrointestinal symptoms and syndromes, were beyond what would be expected in widely inactive Crohn’s disease. On the basis of his medical and personal history since infancy, physical examination, pattern of symptoms, normal laboratory results, absence of any alarm features [8], and a detailed discussion with the informed patient about the course and severity of his symptoms and experiences, we attributed his symptoms to autonomic nervous system dysregulation with disturbance of the gut–brain axis (IBS-D) and CFS.

### Therapeutic approach

According to our hypothesis, the interventions were primarily designed to stabilize the autonomic nervous system of the patient. They included establishing a trusting relationship between patient and healthcare provider and, on this basis, explaining to the patient the probable causes of his condition and the origins of his experienced symptoms in detail. Effective symptom management should demonstrate to him that the hypotheses are correct.

For symptom control, loperamide was substituted with a tincture of opium (an oral solution of anhydrous morphine 10 mg/mL extracted from *Papaver somniferum* L., succus siccus, Dropizol^®^), which, in our experience, is more effective in reducing intestinal motility. Owing to this advantage, opium tincture has been receiving increasing attention in recent literature for the treatment of chronic functional diarrhea [66–69]. The extract contains minor amounts of codeine (methyl morphine) and thebaine (dimethyl morphine), as well as the isoquinoline-derived alkaloids papaverine and noscapine [66, 70].

After oral morphine administration, rapid intestinal absorption occurs, with peak plasma levels reached within 30–90 minutes and an average elimination half-life of 1.4–3.4 hours [69]. Morphine is predominantly biotransformed in the liver to two main metabolites: morphine-3-glucuronide (M3G), a µ-opioid receptor antagonist, and the µ-opioid receptor-active morphine-6-glucuronide (M6G), resulting in a relatively low average morphine bioavailability of 35% [69, 71, 72]. The area under the plasma concentration–time curve (AUC) for M6G has been reported to be 10 times larger than that of morphine [73], suggesting that M6G significantly contributes to morphine’s antidiarrheal effect [69]. In comparison, the plasma half-life of orally administered loperamide (9–14 hours) tends to be higher than that of the active morphine metabolite M6G (9–10 hours); however, its bioavailability may be two orders of magnitude lower, at 0.3% compared with 35% for morphine [69]. Both the higher bioavailability of morphine after oral administration and the accumulation of high M6G plasma levels may explain the efficacy of opium tincture over loperamide in the treatment of chronic diarrhea. An additional antidiarrheal effect may be attributed to codeine [69], which is present in minor quantities in opium tincture. Although codeine appears to be six to seven times less potent at the µ-opioid receptor than morphine [74], its potency is still higher than that of the synthetic opiates loperamide and diphenoxylate [66]. Approximately 10% of orally ingested codeine is converted to morphine, primarily in the liver [74], and subsequently to M3G and the µ-opioid receptor-active metabolite M6G.

The manufacturer’s recommended dose of morphine equivalents for adults with chronic diarrhea is 2.5–5.0 mg two to three times a day; however, the dosage should be customized and fine-tuned to optimize results. Morphine equivalent doses of 1.0–10 mg four times a day [66], 1.5–10 mg four times a day [67], and up to 10 mg in a single dose with a maximum total daily dose of 60 mg [69] have been suggested. Our patient started with three drops of opium tincture twice a day (a total daily dose of 3 mg morphine) and gradually increased the dosage until the desired antidiarrheal effect was achieved. The patient was also advised to take one capsule containing 80 mg of *Lavandula aspic* L. essential oil twice a day (brand name Lasea^®^), which is known for its relaxing and soothing effects. It has proven efficacious in several randomized controlled trials for treating sleep problems and anxiety [75–77]. The patient continued taking 6 g of cholestyramine per day. Ramipril 5 mg once a day was eventually replaced with a combination of 16 mg candesartan cilexetil and 5 mg amlodipine. With a 42-cm ileal resection, our patient was at risk of vitamin B12 malabsorption, which is why we advised him to continue taking methyl cobalamin 500 µg orally disintegrating tablets (ODT) twice a week.

Before presenting to our outpatient clinic, our patient had tried various probiotic preparations containing *Lactobacillus* spp. and *Bifidobacterium* spp., but these did not have the desired positive effect on his intestinal complaints.

Since 1981, the patient has followed a trial and error-based vegetarian diet, which was later maintained for ethical reasons as well. Dietary restrictions based on subjective intolerances included, among others, meat, fish, poultry, cow’s milk, lactose-free milk, ice cream, cornflakes, eggs, hard cheeses, deep-fried foods, hot spices (such as pepper and chili), raw vegetables, tomatoes, apples, citrus fruits, pineapple, rhubarb, acidic fruit juices, and soft drinks, as they presumably caused bowel problems.

We suggested a general reduction of stress to stabilize the disturbed autonomic nervous system and gut–brain axis. Suggestions included maintaining regular meal and sleep timing, such as taking dinner early and in moderate quantities to improve the quality and duration of sleep, and avoiding eating immediately before physical exercise. Regarding diet, we recommended a “back to normal” approach. Only raw food, cruciferous vegetables, and wholegrain bread should be avoided, as they may cause bloating and increase stool frequency. We considered regular physical activity to be a key element in managing IBS. However, since our patient tended to exercise with high tension and self-expectation, which triggered vegetative symptoms, we advised him to perform only moderate physical exercise and to monitor his heart rate, keeping it ≤ 130 bpm. The aim of this energy management, known as pacing, is to avoid overloading and excessive stress in everyday life by adhering to the individual stress limits set by the condition. Furthermore, we suggested that he learns relaxation techniques such as yoga or autogenic training and integrates them into his daily lifestyle.

### Further course of symptoms and patient´s perspective

Our patient reported an immediate and dramatic improvement in both the frequency and severity of diarrhea after increasing the daily dose of opium tincture from three to six drops twice a day (a total daily dose of 6 mg morphine) while simultaneously discontinuing loperamide. The number of BMs decreased from four to six each day to an average of two per day, and in the two following years, only one or two episodes of diarrhea occurred on 2–3 days per month (Tables 1, 2). Along with the reduction in diarrhea, the intermittent abdominal pain notably improved. General fatigue, along with flu-like symptoms, headache, and brain fog, which had persisted for 2–3 weeks every month before our interventions, was soon reduced to 1 week in a 3-month period and was less severe. The associated insomnia was reduced to about 1 week each month, while hip joint pain, urinary urgency, cold extremities, disturbed temperature regulation, and tinnitus remained unchanged initially. Our patient reported the almost immediate reestablishment of social contacts and, after a few months, the ability to undertake long-distance travel and extensive but heart rate-controlled cycling tours, both of which had previously been impossible for several years. After 2–3 hours on flat terrain (road bike) or about 1 hour on inclines (mountain bike), slight bloating sometimes occurred, which subsided after 1–2 hours, but there was no increase in BMs or diarrhea.

Our patient’s condition improved even further from early 2024 (2 years after the beginning of our multimodal treatment) and has stabilized at a good level since then (reported 2.5 years after the start of treatment) over the following 6 months (Tables 1, 2). He attributed the further improvement to the intake of glutamine (10 g twice a day). In patients with IBS-D, increased intestinal permeability might be due to decreased glutamine synthetase levels [78]. In a randomized, double-blind, placebo-controlled, multicenter study including patients with PI-IBS and intestinal hyperpermeability, L-glutamine (5 g three times a day) led to a significant reduction in all important IBS-related endpoints [79]. While flu-like symptoms continued to be felt for about 1 week every 6 months, general fatigue subsided along with abdominal pain, brain fog, dizziness, poor memory, and extreme tiredness. Bloating, headache, temperature dysregulation, tinnitus, and insomnia had clearly lessened. Diarrhea and bloating occasionally occurred after acute stress or physical exertion, but to a much lesser extent. Arthralgia had almost completely disappeared after a total period of 3 years, even without drug therapy with anti-inflammatory painkillers. Low ambient temperatures and physical exertion no longer necessarily led to the development of flu-like symptoms with the described accompanying complaints. The CDAI score [32] was 87 points in June 2024 (weight 81 kg, hematocrit 43.7%), compared with 183 points at the time of our first intervention.

From the patient’s perspective, there was a life before the diagnosis in February 1981 at the age of 23, and another life afterwards. This sentiment was echoed by the surgical ward physician 6 weeks after ileocecal resection, when the patient was discharged on elemental feed, with persistent diarrhea and 15 kg underweight. The physician said, “Say goodbye to your previous life. Stop studying, sit on a deckchair in the garden, and apply for early retirement. Expect further surgeries every two years as long as you still have intestines.” At that time, the patient felt that Crohn’s disease was something new and rare, and that the treating physicians did not really know how to handle his condition. Medical care seemed more focused on healing the extensive scars extending downwards from below the costal arches: one on the right side from iliopsoas abscess drainage, and the other in the center from the ileocecal resection.

After rehabilitation, he decided to rebuild his body by taking up karate. A few years later, he obtained a PhD in chemistry and worked in demanding academic positions with 12-hour days. He is grateful that his life turned out the way it did, despite the unfavorable prognosis at the time and the many challenging days and weeks along the way.

Yet, over the past four decades, diarrhea has been with him like a faithful companion, although CD was repeatedly diagnosed as being in remission. This raised questions and necessitated meticulous planning and coordination of all work processes, meetings, presentations, and other work-related activities, medical appointments, recreational activities, social engagements, and holidays. The morning hours until 10 a.m. were particularly challenging, as urgent bowel movements were common, even without diarrhea. During this time, however, he was required to lead team meetings for ongoing research programmes. Additionally, throughout the 1990s, his role as a quality manager at a food testing facility with 220 employees demanded constant availability. A particular challenge emerged in 2006 when he assumed management of a research unit at a European Reference Laboratory. Throughout his career, only his closest laboratory assistants were privy to his health issues.

During medical consultations following regular colonoscopies at various hospitals, he received no explanation for his persistent diarrhea. Responses such as “that shouldn't really be happening” or “we still know too little about this rare condition” were perceived as unsympathetic, inadequate, and disappointing, leaving him with a sense of abandonment. Therefore, he wished for more robust medical intervention but was left to manage alone. He explored virtually every option that presented itself, even turning to expensive “healers” twice in vain. He thought food utilization was inadequate or several small portions taken throughout the day as recommended did not last. Nevertheless, he had to maintain peak mental performance for his demanding research leadership role.

Between 2012 and 2016, escalating envy-driven bullying compounded the challenges of his working life. As the symptoms increased in number and worsened in early 2017, the carefully maintained façade began to disintegrate. Peculiarly, he now seemed to contract the flu bimonthly, enduring 2–3 weeks of diarrhea, abdominal pain, and an unprecedented level of fatigue. Resorting to a restricted diet of oatmeal, potatoes, and select vegetables, he attempted to minimize appointments. Had the constant stress further exacerbated the strain on his compromised intestines? Like the proverbial frog in boiling water, had he been oblivious to the gradually worsening conditions? After all these years, was he finally confronting a relapse of Crohn’s disease? As bullying intensified and symptoms deteriorated, he was ultimately compelled to take sick leave in July 2017.

His condition took another massive turn for the worse in March 2019 after a severe 3-week bout of infectious gastroenteritis. The following 3 years were marked by spending two-thirds of his life either in bed or at rest. Exposure to low temperatures, such as during a walk or bike ride, or engaging in physical exercise, would trigger debilitating episodes lasting 2–3 weeks. These phases were marked by a sense of malaise, cold extremities, diarrhea up to four times a day with bloating and abdominal pain, profound fatigue, extreme tiredness, and headaches preceding bowel movements. Additionally, he experienced cognitive difficulties, including impaired focus and word recall. His daily functional window was reduced to a mere 1–2 hours in the late morning, during which he could manage housework or nearby shopping, but these activities would leave him exhausted for the remainder of the day. Returning to work was unthinkable, and all social contacts had ceased. Then, in November 2020, severe hip pain developed, first unilaterally and later bilaterally. By September 2021, after weeks of struggling to walk and contemplating hip replacement, a specialist reviewed his X-rays and MRI scans, diagnosing mild hip osteoarthritis consistent with his age but unable to account for the severity of his pain. Further consultations with specialists in gastroenterology, internal medicine, nutrition, and even psychosomatics (as he felt stress aggravated many of his symptoms) yielded no conclusive explanation for his symptom pattern. Now in early retirement, barely able to eat, walk, or sleep properly, and incapable of engaging in any activities, he felt hopeless, with no light at the end of the tunnel.

Utterly disheartened but persistently encouraged by his wife, a physician at the same university hospital, he resolved to consult a specialist in internal medicine, gastroenterology, and naturopathy at the Centre for Complementary Medicine at the University of Freiburg’s Faculty of Medicine in February 2022, as a last resort. Remarkably, the half-hour consultation was phenomenal and completely turned the tide. In an atmosphere of mutual partnership, empathy, and understanding, he received, for the first time in four decades, a plausible explanation for his persistent diarrhea and the numerous other more recent symptoms. The immense relief and calming, positive effect of finally being so well informed after a long and arduous journey cannot be overstated. Suddenly, his condition and symptoms made perfect sense. He could scarcely have envisaged that his health issues did not stem from remitted Crohn’s disease and recurring flu-like episodes due to a compromised immune system, as previously suggested by some specialists. Instead, he discovered that, in addition to Crohn’s disease, he had developed diarrhea-predominant irritable bowel syndrome, exacerbated by the severe gastrointestinal infection in spring 2019, to which a chronic fatigue syndrome then became associated. He learned that his flu-like symptoms could be reinterpreted as consequences of vegetative nervous system dysfunction, brought on by chronic life stress. This new understanding provided invaluable insight into the origins and interrelations of his symptoms. Nevertheless, during that 30-minute patient-centered consultation, he could not have anticipated the remarkable success of the proposed interventions that would soon follow.

Replacing loperamide with opium tincture, having an early dinner, and managing his physical activities led to an immediate and substantial improvement in his quality of life within just a few days. Diarrhea episodes reduced dramatically from up to four times a day to only once or twice a day on just two to three days a month. The frequency and intensity of bloating, abdominal pain, headache, brain fog, dizziness, impaired memory, extreme tiredness, and insomnia also significantly decreased over the following months, although strong bilateral hip joint pain persisted. For the first time in 5 years, he was able to undertake long-distance flights to North America and visit family and friends in the Asia–Pacific region.

L-glutamine further enhanced and stabilized his condition. Within 4–6 weeks, general fatigue, brain fog, dizziness, impaired memory, and extreme tiredness had largely dissipated. Bloating still occurred occasionally after acute stress or physical exertion but was mild and mostly pain-free. With few exceptions, his sleep duration normalized to 6–7 hours. His diet expanded to include, for the first time in 43 years, wholegrain bread, hard cheese, and French fries. He tolerated up to 90 minutes at an ice rink without subsequent flu-like or gastrointestinal symptoms, and similarly fared well after an intercontinental flight. However, reducing his daily intake of L-glutamine from 20 to 10 g on a trial basis resulted in bloating and general discomfort, which resolved within a few days upon resuming the 20 g dose. Currently, even without painkillers, he experiences only minor and diminishing one-sided hip joint pain. Instead of dressing excessively warm to prevent shivering, as had been necessary even on warm summer days for the past 5 years, he now enjoys wearing T-shirts again in temperatures above 20 °C. He feels more resilient and is able to work intensively for 8 hours a day without gastrointestinal complaints. Coming to terms with his medical history has brought a deep sense of relief and optimism for the future. In essence, thanks to a skilled complementary medicine intervention and comprehensive education, he finally feels liberated from the passive “victim role” and has regained his life.

## Discussion and conclusion

Our case presentation illustrates several key aspects. Firstly, it shows that functional diseases (IBS) may occur simultaneously with severe organic diseases (IBD) and that differentiation can be challenging and may be overlooked. Gastroenterologists may have overly focused on endoscopy, while psychosomatic specialists might have been uncertain about their ability to assist this patient. Secondly, it exemplary shows the bidirectional interrelation between IBS, CFS, and fibromyalgia/somatoform pain syndrome. The organic disease (IBD) and IBS presented first, then CFS occurred during a period of massive psychological stress, with the pain syndrome subsequently settling on top. Resolution of symptoms occurred in the same order: IBS improved first, followed by CFS, and finally, the pain disappeared. It is known that the combination of IBS, CFS, and fibromyalgia/chronic pain syndrome is interlinked and indicative of a more severe course than any of these conditions alone [80]. Not all fibromyalgia criteria were met (only hip pain and fatigue), but in the absence of explanatory organic findings, the hip pain can most likely be interpreted as a somatoform symptom. Thirdly, the case report demonstrates the importance of understanding and explaining the patient’s symptoms, even though some, such as deterioration after exposure to cold air, are unusual. An in-depth history-taking, the uncovering of traumatic life experiences since early childhood, and knowledge of autonomic nervous system regulation, temperature regulation, and psycho-neuro-immunological connections proved instrumental in explaining these symptoms. The German S3 Guideline on Irritable Bowel Syndrome [81] emphasizes the importance of properly explaining the nature of functional connections such as the gut–brain axis. However, the expertise of how to explain these connections is beyond the guideline and, to our knowledge, is not an integrative part of the curriculum for medical students, at least in Germany. Furthermore, this case illustrates that a transparent, comprehensible explanation of symptoms, combined with effective symptom management, can lead to rapid and sustained improvement even in a severe, longstanding case of IBS lasting decades and CFS persisting for 3 years. While self-management strategies (such as paced physical training, relaxation techniques, structured daily routines, and dietary adjustments) were beneficial and are recommended in treatment guidelines for IBS [81] and CFS [82–84], they alone would not explain the rapid improvement starting already a few days after the initial visit. We assume that physical training and relaxation techniques needed more time to improve our patient’s physical fitness, self-trust, and autonomic regulation. Further important factors contributing to the favorable outcome included, in our view, the patient’s excellent motivation, intellectual capacity to understand the explanations, proactive coping strategies, and trust in both the attending specialist and the treatment process.

## Data Availability

Not applicable.

## Abbreviations

AUC: Area under the curve
BM: Bowel movement
BRS: Baroreceptor sensitivity
C6G: Codeine-6-glucuronide
CD: Crohn’s disease
CDAI: Crohn’s Disease Activity Index
CFS: Chronic fatigue syndrome
CFU: Colony-forming unit
CNS: Central nervous system
CRP: C-reactive protein
DGBI: Disorder of gut–brain interaction
ENS: Enteric nervous system
ESR: Erythrocyte sedimentation rate
FM: Fibromyalgia
GMC: General medical condition
HPA: Hypothalamic–pituitary–adrenal
HR: Heart rate
IBD: Inflammatory bowel disease
IBS: Irritable bowel syndrome
IBS-D: Irritable bowel syndrome with predominant diarrhea
ICR: Ileocecal resection
M3G: Morphine-3-glucuronide
M6G: Morphine-6-glucuronide
MGB: Microbiota–gut–brain
MRI: Magnetic resonance imaging
OGD: Esophagogastroduodenoscopy
PI-IBS: Post-infectious irritable bowel syndrome
PNS: Parasympathetic nervous system
U/S: Ultrasound imaging (sonography)
UC: Ulcerative colitis
UZN: Uni-Zentrum Naturheilkunde (University Center for Complementary Medicine)
